# Characterization and functional analysis of seven flagellin genes in *Rhizobium leguminosarum *bv. *viciae*. Characterization of *R. leguminosarum *flagellins

**DOI:** 10.1186/1471-2180-10-219

**Published:** 2010-08-17

**Authors:** Dinah D Tambalo, Denise E Bustard, Kate L Del Bel, Susan F Koval, Morgan F Khan, Michael F Hynes

**Affiliations:** 1 Department of Biological Sciences, University of Calgary. 2500 University Drive NW. Calgary, AB, T2N 1N4, Canada; 2 Department of Microbiology and Immunology, University of Western Ontario, London, Ontario, N6A 5C1, Canada; 3 Mass Spectrometry Facility, Faculty of Medicine, University of Calgary. 2500 University Drive NW. Calgary, AB, T2N 1N4, Canada

## Abstract

**Background:**

*Rhizobium leguminosarum *bv. *viciae *establishes symbiotic nitrogen fixing partnerships with plant species belonging to the Tribe Vicieae, which includes the genera *Vicia, Lathyrus, Pisum *and *Lens*. Motility and chemotaxis are important in the ecology of *R. leguminosarum *to provide a competitive advantage during the early steps of nodulation, but the mechanisms of motility and flagellar assembly remain poorly studied. This paper addresses the role of the seven flagellin genes in producing a functional flagellum.

**Results:**

*R. leguminosarum *strains 3841 and VF39SM have seven flagellin genes (*flaA*, *flaB, flaC, flaD, flaE, flaH*, and *flaG*), which are transcribed separately. The predicted flagellins of 3841 are highly similar or identical to the corresponding flagellins in VF39SM. *flaA, flaB, flaC*, and *flaD *are in tandem array and are located in the main flagellar gene cluster. *flaH *and *flaG *are located outside of the flagellar/motility region while *flaE *is plasmid-borne. Five flagellin subunits (FlaA, FlaB, FlaC, FlaE, and FlaG) are highly similar to each other, whereas FlaD and FlaH are more distantly related. All flagellins exhibit conserved amino acid residues at the N- and C-terminal ends and are variable in the central regions. Strain 3841 has 1-3 plain subpolar flagella while strain VF39SM exhibits 4-7 plain peritrichous flagella. Three flagellins (FlaA/B/C) and five flagellins (FlaA/B/C/E/G) were detected by mass spectrometry in the flagellar filaments of strains 3841 and VF39SM, respectively. Mutation of *flaA *resulted in non-motile VF39SM and extremely reduced motility in 3841. Individual mutations of *flaB *and *flaC *resulted in shorter flagellar filaments and consequently reduced swimming and swarming motility for both strains. Mutant VF39SM strains carrying individual mutations in *flaD, flaE, flaH*, and *flaG *were not significantly affected in motility and filament morphology. The flagellar filament and the motility of 3841 strains with mutations in *flaD *and *flaG *were not significantly affected while *flaE *and *flaH *mutants exhibited shortened filaments and reduced swimming motility.

**Conclusion:**

The results obtained from this study demonstrate that FlaA, FlaB, and FlaC are major components of the flagellar filament while FlaD and FlaG are minor components for *R. leguminosarum *strains 3841 and VF39SM. We also observed differences between the two strains, wherein FlaE and FlaH appear to be minor components of the flagellar filaments in VF39SM but these flagellin subunits may play more important roles in 3841. This paper also demonstrates that the flagellins of 3841 and VF39SM are possibly glycosylated.

## Background

Motility is an important property of bacteria that enables them to move towards favorable growth conditions and away from detrimental conditions. Most bacteria move through the use of flagella. A bacterial flagellum consists of three distinct regions: the basal body, the hook, and the filament [1]. Flagellar assembly and motility are well-understood in enteric bacteria, particularly *Escherichia coli *and *Salmonella*. The flagellar filament of *E. coli *is a helical arrangement of as many as 20,000 flagellin subunits, whose molecular weight is approximately 50 kDa [1,2]. Whereas the *E. coli *flagellar filament consists of one type of flagellin [3,4], the presence of more than one flagellin type has been reported for a few soil bacteria, including *Sinorhizobium meliloti*, *Rhizobium lupini*, and *Agrobacterium tumefaciens *[5-10]*. S. meliloti *and *A. tumefaciens *assemble their flagellar filaments from four closely related flagellin subunits (FlaA, FlaB, FlaC, and FlaD) while *R. lupini *flagella consist of three flagellin subunits (FlaA, FlaB, and FlaD). For these soil bacteria, FlaA is the principal flagellin subunit of the flagellar filament while the other subunits play minor roles.

The flagellar filament is a highly conserved structure in terms of amino acid composition, subunit domain organization of the flagellin monomers, and the symmetry and mode of assembly [11,12]. The quaternary structure of the flagellar filament has been divided into four structural domains, domain 0 (D0) to domain 3 (D3), and the amino acid residues of the flagellin protein have been assigned to these domains [13-17]. Domains D0 and D1, which are found in the filament core, correspond to the amino and carboxy terminal residues. Domains D2 and D3, the outer region of the filament, consist of the flagellin central residues. The amino acid sequences corresponding to domains 0 and 1 are highly conserved across different bacterial strains [14,18], and were shown to be essential in the polymerization of bacterial flagellar filaments [19]. Domains D2 and D3, on the other hand are considerably variable in amino acid composition and are generally not well-aligned [18]. Domain D3 of the filament contributes to filament stability [16] but it can be deleted or reduced in size without severely impairing filament assembly and function [16,20-22].

Flagellar filaments are traditionally classified as either "plain" or "complex". Plain filaments are often found in enterobacteria, such as *Salmonella typhimurium *and *E. coli *[23,24]. These filaments have a smooth surface and are able to change from left- to right-handedness or from a counterclockwise to a clockwise direction of rotation [5]. A few soil bacteria such as *Pseudomonas rhodos *[25], *R. lupini *[24,26] and *S. meliloti *[26] are equipped with one or more complex flagella. Studies have shown that transmission electron microscopy can be used to differentiate between plain and complex flagella [24,27]. Complex flagellar filaments have a distinct ridging pattern while plain filaments appear thinner and have little to no visible external pattern. The complex filaments are also more rigid and more brittle than the plain filament. It is thought that increased rigidity is favorable for motility in viscous environment such as in the soil biotope [27].

To date, little is known about the flagellar filament of *Rhizobium leguminosarum *bv. *viciae*. A previous study has shown that the movement of *R. leguminosarum *bv. *viciae *strain 3841 is propelled by one or two subpolar flagella [28]. The same study has also suggested that the flagella rotate in a unidirectional pattern and the direction of movement is changed by modulating the rotary speed. In this paper, we characterize the genes encoding the seven flagellin subunits in *R. leguminosarum *bv. *viciae*. We have conducted sequence analysis, as well as mutational and transcriptional studies to determine the roles of the flagellin genes in flagellar assembly and function for the sequenced strain 3841 and our laboratory strain VF39SM. We have studied the flagellin genes in parallel in both strains because the two strains exhibit differences in pattern of flagellation (see below) and also in swarming motility (below and [29]).

## Methods

### Bacterial strains, plasmids, and growth conditions

The bacterial strains and plasmids used in this study are shown in Table 1. *R. leguminosarum *and *E. coli *strains were grown in TY medium [30] and LB medium [31], respectively. The concentrations of antibiotics used to grow *R. leguminosarum *were streptomycin (Sm) 500 μg/ml, gentamicin (Gm) 30 μg/ml, neomycin (Nm) 100 μg/ml, spectinomycin (Sp) 100 μg/ml, and tetracycline (Tc) 5 μg/ml. *E. coli *strains were grown in the following antibiotic concentrations: ampicillin (Ap) 100 μg/ml, kanamycin (Km) 50 μg/ml, gentamicin (Gm) 15 μg/ml, and tetracycline (Tc) 10 μg/ml.

**Table 1:** Bacterial strains and plasmids used in the study.

Strains and Plasmids	Relevant characteristics	Source or Reference
*Escherichia coli *strains		
DH5α	*endA1*, *hsdR17*, *supE44*, *thi-1*, *recA1*, *gyrA96*, *relA1*,(*argF-lacZYA*), U169, φ 80d*lacZ *ΔM15	Invitrogen
S17.1	Sp^r^. RP4 *tra *region, mobilizer strain	[69]
*Rhizobium leguminosarum *strains		
3841	biovar *viciae*, JB300 derivative, *Sm*^*r*^	[70]
VF39SM	biovar *viciae*, *Sm*^*r*^	[71]
VF39SM *flaA*^-^	VF39SM *flaA*^-^, *Sm*^*r *^,*Nm*^*r*^	This work
VF39SM*flaA*^+^	VF39SM*flaA*^- ^complemented with *flaA, Sm*^*r*^, *Nm*^*r*^,*Gm*^*r*^	This work
3841 *flaA*^-^	*gusA*-*Nm *cassette insertion in 3841 *flaA*, *Sm*^*r*^, *Nm*^*r*^	This work
3841*flaA*^+^	3841*flaA*^- ^complemented with *flaA, Sm*^*r*^, *Nm*^*r*^,*Gm*^*r*^	This work
VF39SM *flaB*^-^	Spectinomycin cassette insertion in VF39SM *flaB*, *Sm*^*r *^,*Sp*^*r*^	This work
3841 *flaB*^-^	Spectinomycin cassette insertion in 3841 *flaB*, *Sm*^*r *^, *Sp*^*r*^	This work
VF39SM *flaC*^-^	*gusA*-*Nm *cassette insertion in VF39SM *flaC*, *Sm*^*r*^, *Nm*^*r*^	This work
3841 *flaC*^-^	*gusA*-*Nm *cassette insertion in 3841 *flaC*, *Sm*^*r*^, *Nm*^*r*^	This work
VF39SM *flaD*^-^	*gusA*-*Nm *cassette insertion in VF39SM *flaD*, *Sm*^*r*^, *Nm*^*r*^	This work
3841 *flaD*^-^	*gusA*-*Nm *cassette insertion in 3841 *flaD*, *Sm*^*r*^, *Nm*^*r*^	This work
VF39SM *flaE*^-^	*gusA*-*Nm *cassette insertion in VF39SM *flaE*, *Sm*^*r*^, *Nm*^*r*^	This work
3841 *flaE*^-^	*gusA*-*Nm *cassette insertion in 3841 *flaE*, *Sm*^*r*^, *Nm*^*r*^	This work
VF39SM *flaH*^-^	Neomycin-resistance cassette insertion in VF39SM *flaH*, *Sm*^*r*^, *Nm*^*r*^	This work
3841 *flaH*^-^	Neomycin-resistance cassette insertion in 3841 *flaH*, *Sm*^*r*^, *Nm*^*r*^	This work
VF39SM *flaG*^-^	Tetracycline-resistance cassette insertion in VF39SM *flaG*, *Sm*^*r*^, *Tc*^*r*^	This work
3841 *flaG*^-^	Tetracycline-resistance cassette insertion in 3841 *flaG*, *Sm*^*r*^, *Tc*^*r*^	This work
3841 *flaA/B/C/D*^-^	3841 strain with mutations in *flaA/B/C/D*, *Sm*^*r*^, *Nm*^*r*^	This work
VF39SM *flaA/B/C/D*^-^	VF39SM strain with mutations in *flaB/C/D*, *Sm*^*r*^, *Nm*^*r*^	This work
VF39SM *flaB/C/D*^-^	VF39SM *flaA/B/C/D *^- ^complemented with *flaA*; *Sm^r^*, *Nm^r^*, *Gm^r^*	This work
3841 *flaB/C/D ^-^*	3841 *flaA/B/C/D *^- ^complemented with *flaA*; *Sm^r^*, *Nm^r ^*, *Gm*^*r*^	This work
*Plasmids*		
pCR2.1-TOPO	Cloning vector, *Amp^r ^*, *Km^r^*	Invitrogen
pJQ200SK	Suicide vector with *sacB *system; *Gm^r^*	[32]
pJQ200mp18	Suicide vector with *sacB *system; *Gm^r^*	[32]
pCRS530	Contains a promoterless *gusA*-*Nm *cassette	[33]
pBSL99	Contains kanamycin-resistance cassette	[36]
pBSIISK+	Cloning vector, *Amp^r^*	Stratagene
pBS::*flaD*3'-Km-*flaA*5'	*flaA*5' fragment (from pCR2.1::*flaA*5') subcloned into pBS::*flaD*3'-Km, *Amp^r ^, Km^r^*	This work
pJQmp18:: *flaD*3'-Km-*flaA*5'	*flaD*3'-Km-*flaA*5' fragment subcloned from pBS::*flaD*3'-Km-*flaA*5' into pJQmp18, *Gm^r ^, Km^r^*	This work
pHP45Ω	Contains omega-spectinomycin cassette; *Sp^r^*	[34]
pHP45:ΩTc	Contains tetracycline-resistance cassette; *Tc^r^*	[35]
pBBR1-MCS5	Broad-host-range cloning vector, *Gm^r^*	[72]
pFus1	pMP220 derivative with promoterless *gusA*, *Tc^r^*	[33]
pBBR1-MCS5:3841*flaA*	Broad-host-range cloning vector containing *flaA *gene (with promoter region) from 3841	This work
pBBR1-MCS5:VF39SM*flaA*	Broad-host-range cloning vector containing *flaA *gene (with promoter region) from VF39SM	This work
pFus1::*flaB*	*flaB *promoter introduced into pFus1, *Tc^r^*	This work

*Ap*^*r *^Ampicillin resistance, *Gm*^*r *^Gentamicin resistance, *Nm*^*r *^Neomycin resistance, *Sm*^*r *^Streptomycin resistance, *Sp*^*r *^Spectinomycin resistance, *Tc*^*r *^Tetracycline resistance

### Recombinant DNA techniques

Recombinant DNA techniques were performed using standard methods [31]. Restriction endonucleases used in this study were purchased from Invitrogen or New England Biolabs and used according to the manufacturer's specifications. DNA fragments were isolated from agarose gels using Qiaquick Gel Extraction kit (Qiagen). Plasmids were isolated from *E. coli *strains using GeneJET™ Plasmid Miniprep kit (Fermentas Life Sciences). Total DNA was isolated from *R. leguminosarum *strains using Aquapure Genomic DNA Isolation kit (Bio-Rad Laboratories). Primers were synthesized by Sigma Genosys (Sigma-Aldrich) and amplification was carried out using a Multi GeneII PCR machine (Labnet International, Inc.). Southern blots were performed using a non-radioactive technique with reagents and protocols supplied by Roche Applied Science.

### Mutagenesis of flagellin genes

The seven *fla *genes were PCR amplified from *R. leguminosarum *using the primers listed in Additional file 1. The PCR products were individually cloned into the vector pCR2.1-TOPO using the TOPO Cloning kit (Invitrogen). The genes were excised from the TOPO vector and then ligated into either pJQ200SK or pJQ200mp18 [32]. The details on constructing the individual *fla *mutants are presented in Additional file 2. Individual mutations in *flaA*, *flaC*, *flaD*, and *flaE *were introduced by inserting a *gusA-Nm^r ^*(CAS-GNm) cassette from pCRS530 [33] into the reading frame of each gene. The *flaB *and *flaG *genes were mutated by inserting a spectinomycin and tetracycline resistance cassette, respectively, from pHP45:Ω [34] and pHP45:Ω-Tc [35]. The *flaH *gene was mutated by inserting a kanamycin-resistance cassette from pBSL99 [36]. The *flaA/B/C/D *genes were mutated by separately amplifying the 5' end of *flaA *plus flanking region (missing the 3' end of *flaA*) and the 3' end of *flaD *plus flanking region (missing the 5' end of *flaD*). The truncated genes were cloned separately into pCR2.1-TOPO and the resulting plasmids (pCR2.1::*flaA*5' and pBS::*flaD*3') were sequenced at the University of Calgary Core DNA Services. The fragment containing the truncated *flaD *gene was subcloned into pBSIISK+ (Stratagene) creating pBS::*flaD*3'. A kanamycin-resistance cassette (Km) from pBSL99 [36] was ligated upstream of the *flaD*3' fragment resulting in the construct pBS::*flaD*3'-Km. The fragment containing the truncated *flaA *gene (from pCR2.1::*flaA*5') was subcloned into pBS::*flaD*3'-Km, upstream of the Km-cassette creating pBS::*flaD*3'-Km-*flaA*5'. A fragment containing the truncated *flaA *gene, kanamycin resistance cassette, and truncated *flaD *gene was subcloned from pBS::*flaD3*'-Km-*flaA*5' into pJQ200mp18 [32] creating pJQmp18::*flaD*3'-Km-*flaA*5'. Each of the mutated gene/s was introduced into the genome of *R. leguminosarum *by homologous recombination. The *flaA/B/C/D *mutants have deletions in the following: *flaA *3' end; *flaB*; *flaC*; and *flaD *5' end. Southern hybridization and/or PCR were performed for each gene to confirm replacement of the wild-type gene with the mutated gene/s.

### Construction of gene fusions and ß-glucuronidase (*gusA*) reporter gene assays

The promoter region of *flaB *was cloned upstream of a promoterless *gusA *gene in pFus1 [33]. The resulting construct was introduced into VF39SM and 3841 by biparental mating. VF39SM and 3841 strains containing the *flaB-gusA *fusion were grown in TY broth for 48 hours at 30°C [33]. β-glucuronidase activity was measured as described by Jefferson *et al. *[37] and modified by Yost *et al. *[38]. The data given are the means of triplicate experiments.

### Swimming motility test

The strains were grown in TY broth for 24 hours. Swimming motility was determined by inoculating the strains into a motility medium (YES) containing the following: 0.3% agar, 0.01% yeast extract, and 1 mM MgSO_4 _[39]. The optical densities (OD600) of the cultures were standardized and equal amounts of inoculum were inoculated into the swimming agar using a fine-point pipette tip. The swimming diameter was measured 3-4 days after inoculation.

### Swarming Motility Test

The swarm assay was performed following the method described by Tambalo *et al. *[29]. Briefly, *R. leguminosarum *wildtype and *fla *mutant strains were grown in TY broth for 24 hours. Equal amounts of inoculum from the TY culture was used to inoculate swarm plates. The plates were incubated at 22°C for two to three weeks and the swarming motility of the *fla *mutants was compared with the wildtype.

### Flagellar filament isolation

Flagellin proteins were isolated from *R. leguminosarum *based on the procedure described by Maruyama *et al. *[40]. Cells were grown in 100 ml of TY broth for 48 hours with slow agitation (50 rpm). The bacterial cells were collected by centrifugation at 12,000 × g for 10 minutes. The pellet was resuspended in 40 mM phosphate buffer. The bacterial cells were vigorously agitated using a vortex to detach the flagella from the cells. The mixture was centrifuged at 12,000 × g for 10 minutes using a Sorval centrifuge. The supernatant was removed and centrifuged again at the same speed and time. The supernatant containing the detached flagella was centrifuged in an ultracentrifuge at 50,000 × g for 2 hours. The pellet was resuspended in 200 μL of 40 mM phosphate buffer.

### Immunoblot

The flagellar protein samples were denatured at 100°C for 5 minutes and then separated on 12% acrylamide SDS-PAGE gel at 200V for 45 minutes. Molecular size markers from Bio-Rad and Fermentas were used. After electrophoresis, the gel was blotted onto a PVDF membrane (Bio-Rad) using the Bio-Rad apparatus and protocol for electrophoretic transfer. The blot was blocked with 10% skim milk solution for 2 hours. After washing with phosphate-buffered saline (PBS) solution, the blot was probed overnight using a polyclonal flagellar antibody raised in a rabbit against isolated flagellar filaments [41]. Protein A-alkaline phosphatase (Sigma-Aldrich) was used as the secondary antibody. The blot was washed with PBS and was developed using NBT/BCIP (Sigma).

### Preparation of samples for tandem mass spectrometry analysis (MS/MS)

The flagellar protein samples were run on a polyacrylamide gel as described above. Staining and destaining of the protein gel were performed following standard protocols [42]. The gel was soaked overnight in a staining solution containing 0.1% Coomassie Brilliant Blue (R-250; Sigma), 40% methanol, and 10% acetic acid. Destaining was done using a solution containing 40% methanol and 10% acetic acid. The bands (between approximately 25-37kDa) were excised and submitted to the Southern Alberta Mass Spectrometry (SAMS) Centre at the University of Calgary for LC-MS/MS analysis. Two bands within the size range were observed in the gel. The two bands were analyzed separately for 3841 and in combination for VF39SM.

The gel slices were rinsed once with HPLC-grade water and then twice with 25 mM ammonium bicarbonate in 50% (v/v) acetonitrile. The gel slices were dehydrated with acetonitrile prior to lyophilization. The dehydrated gel was resuspended in 25 mM ammonium bicarbonate (pH8.0) and samples were digested with trypsin. The peptides were extracted from the gel using 1% formic acid in 50% acetonitrile. The extracts were reduced to dryness and then reconstituted in mobile phase of the buffer (3% acetonitrile with 0.2% formic acid) for liquid chromatography.

### Tandem mass spectrometry analysis (MS/MS)

The digests were analyzed using an integrated Agilent 1100 LC-Ion-Trap-XCT-Ultra system (Agilent Technologies, Santa Clara, CA), which has an integrated fluidic cartridge for peptide capture, separation, and nano-spraying (HPLC Chip). The injected samples were trapped and desalted for 5 minutes using a pre-column channel (40-nl volume; Zorbax 300 SB-C_18_) with an auxiliary pump that delivers 3% acetonitrile and 0.2% formic acid at a flowrate of 4 μl/minute. The peptides were reverse-eluted from the trapping column and separated on a 150 mm-long analytical column (Zorbax 300SB-C_18_) at a flowrate of 0.3 μl/minute. The peptides were eluted using a 5-70% (v/v) acetonitrile gradient in 0.2% (v/v) formic acid over a period of 10 minutes. The MS/MS spectra were collected by data-dependent acquisition, with parent ion scans of 8100 Th/s over *m*/*z *400-2,000. MS/MS scans at the same rate over *m*/*z *100-2200.

### Mass Spectrometry Data Analysis

DataAnalysis software for the 6300 series ion trap, v3.4 (build 175) was used to extract the peak-list data. The MS/MS data were analyzed using Mascot v2.1 (Matrix Science, Boston, MA) with the following parameters: 1.6 Da precursor ion mass tolerance, 0.8 Da fragment ion mass tolerance, and one potential missed cleavage. A protein database for *R. leguminosarum *3841 was obtained from the Wellcome Trust Sanger Institute website ftp://ftp.sanger.ac.uk/pub/pathogens/rl/ and was deposited in Mascot. The deposited *R. leguminosarum *3841 protein database was used for database searching to identify the proteins present in the flagellar preparations. A cut-off score (*p *= 0.05) of 31 was used for all peptides and since the flagellins of *R. leguminosarum *are highly homologous, we required at least one unique peptide for a flagellin protein to be considered a match. We also determined the relative abundance of the flagellin proteins based on the exponentially modified protein abundance index (emPAI) values, which were automatically generated using MASCOT analysis. The emPAI value is based on the correlation of the observed flagellin peptides in the MS/MS analysis and the number of observable peptides (obtained by *in silico *digestion) for each flagellin protein [43,44].

### Glycoprotein staining

Flagellar preparations from VF39SM and 3841 were run on 12% acrylamide at 200V for 1 hour and 15 minutes. Glycosylation of flagellin subunits was determined using a Pro-Q Emerald 300 glycoprotein gel stain kit (Molecular Probes) following the manufacturer's instructions. After glycoprotein staining, the total protein was visualized by staining the gel with 0.1% Coommassie Blue.

### Transmission electron microscopy

Transmission electron microscopy was performed by slightly modifying the procedure used by Miller *et al. *[28]. The *R. leguminosarum *wildtype and *fla *mutant strains were grown on TY plates at 30°C for 48 hours. A culture suspension was prepared using sterile double distilled water. A formvar carbon-coated grid was placed on top of a cell suspension drop for 3 minutes and excess liquid was removed. Staining was performed using 1% uranyl acetate for 30 seconds. Samples were observed using a Philips 410 transmission electron microscope or a Hitachi-7650 transmission electron microscope with images taken with an AMT Image capture Engine. The length of the flagellar filaments formed by the wildtype and mutant strains was measured using Scion Image http://www.scioncorp.com/.

## Results and Discussion

### Characterization of flagellin genes in *R. leguminosarum *

**Figure 1:**
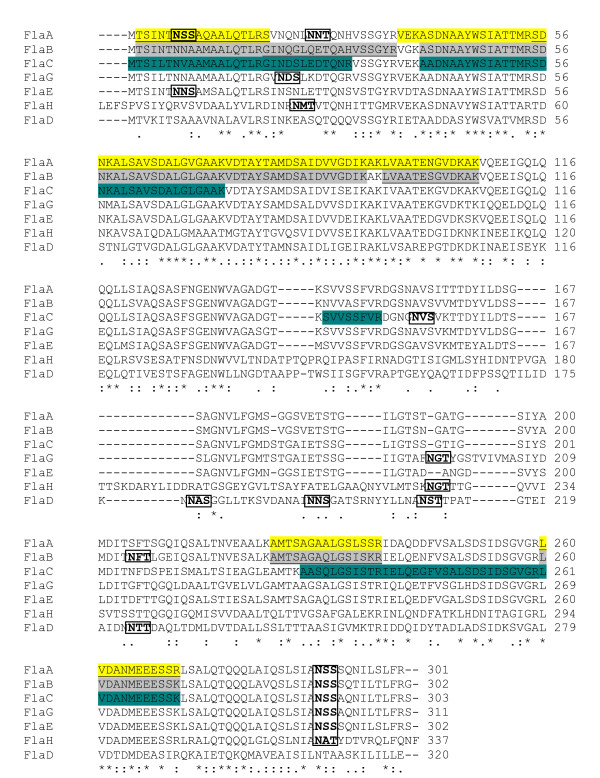
**Sequence alignment of the seven flagellin subunits of *R. leguminosarum *bv. *viciae *strain 3841**. Asterisks represent conserved residues; colons represent conserved substitutions; dots represent semi-conserved substitutions. The tryptic peptides detected in the upper band for 3841wt flagellar preparations are highlighted. FlaA peptides are highlighted in yellow; FlaB peptides are highlighted in gray; FlaC peptides are highlighted in teal. The peptides unique for the flagellin subunit are underlined. The glycosylation signals are in boxes. The sequence coverage of FlaA, FlaB, and FlaC are 44%, 37%, and 31%, respectively.

**Figure 2:**
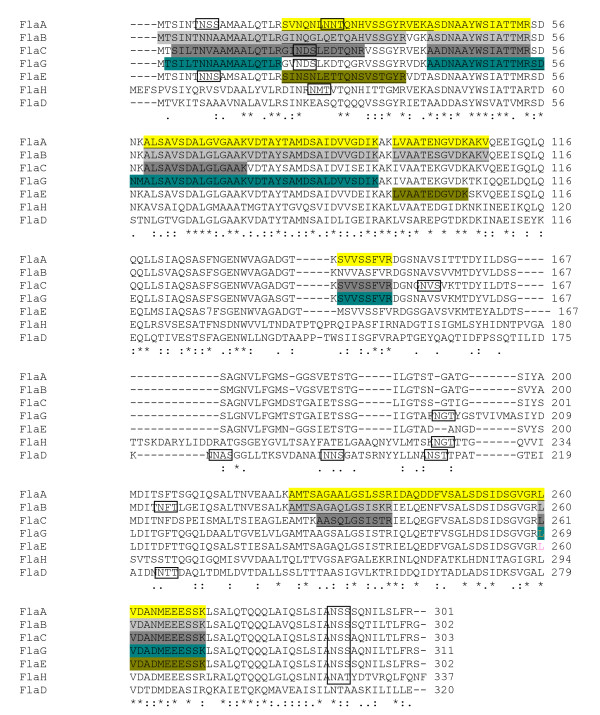
**Alignment of *R. leguminosarum *VF39SM flagellin amino acid sequences**. Asterisks represent conserved residues; colons represent conserved substitutions; dots represent semi-conserved substitutions. The tryptic peptides detected in the flagellar samples by tandem mass spectrometry are highlighted. FlaA peptides are highlighted in yellow; FlaB peptides are highlighted in light gray; FlaC peptides are highlighted in dark gray; FlaG peptides are highlighted in teal; FlaE peptides are highlighted in moss green. The peptides unique for each flagellin are underlined. The glycosylation signals are in boxes. The sequence coverage of FlaA, FlaB, FlaC, FlaG, and FlaE are 46%, 43%, 29%, 28%, and 18%, respectively.

There are seven flagellin (*fla*) genes (*flaA *RL0718*, flaB *RL0719*, flaC *RL0720*, flaD *RL0721*, flaE *pRL110518*, flaH *RL3268, and *flaG *RL4729) in the genome of *R. leguminosarum *bv. *viciae *strain 3841 [45]. Sequence analysis and transcriptional studies indicate that all of the seven flagellin genes are transcribed separately as monocistronic genes. Six flagellin genes (*flaA/B/C/D/H/G*) are found on the chromosome, with *flaA/B/C/D *located within the major chemotaxis and motility gene cluster [28] while *flaE *is encoded on plasmid pRL11. The sizes of the predicted encoded proteins are: FlaA, 301 amino acids; FlaB, 302 amino acids; FlaC, 303 amino acids; FlaD, 320 amino acids; FlaE, 302 amino acids; FlaH, 337 amino acids, and FlaG, 311 amino acids, respectively. The sizes of these flagellin subunits are smaller than the flagellin proteins of *S. meliloti *(321 to 401 amino acids) [46,47] and *R. lupini *(410-430 amino acids) [5]. The predicted molecular masses of the proteins are: FlaA-31 kDa; FlaB-31 kDa; FlaC-31 kDa; FlaD-34 kDa; FlaE-31; kDa; FlaH-36 kDa; FlaG-32 kDa. Our group has also determined the sequences of the flagellin genes of *R. leguminosarum *strain VF39SM (Genbank accession number GU071045 for *flaA/B/C/D*; GU071046 for *flaE*; GU071047 for *flaH*; and GU071048 for *flaG*) and found that the predicted flagellin subunits of this strain are 99% to 100% identical to the corresponding flagellins in 3841. All of the flagellin proteins of *R. leguminosarum *exhibit conserved residues at the amino and carboxy-terminal ends (Fig. 1 and 2). The central regions of the proteins, on the other hand, contain the highest variability. In terms of flagellin sequence similarity, FlaA/B/C/E/G are highly similar, exhibiting 86-93% similarity to each other. The other two flagellins, FlaD and FlaH, are more distant, and respectively share 62% and 64% similarity with FlaA.

### Ultrastructure of the flagellar filament of *R. leguminosarum*

**Figure 3:**
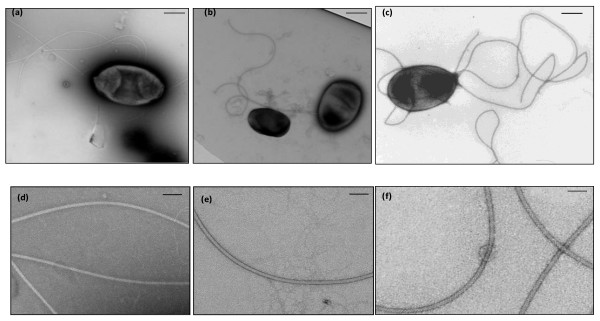
**Electron micrographs of *R. leguminosarum *and *S. meliloti *1021 flagellar filaments stained with 1% uranyl acetate**. (a) VF39SM is peritrichously flagellated; (b) 3841 has a subpolar flagellum; (c) *S. meliloti *1021 is peritrichously flagellated. The flagellar filaments of (d) VF39SM and (e) 3841 appear to have a smooth surface and lack the ridging pattern observed on the surface of the complex flagella formed by (f) *S. meliloti *1021. Bars: 500 nm for a, b and c; 100 nm for d, e and f.

Electron microscopy work confirmed that *R. leguminosarum *bv. *viciae *strain 3841 is subpolarly flagellated [28], while strain VF39SM is peritrichously flagellated, exhibiting 4-7 flagella per cell (Fig. 3). The flagellar filaments of strains 3841 and VF39SM were around 5 μm in length and averaged 18 nm and 17 nm in width, respectively. The surface of the filaments appeared smooth (Fig. 3c and 3d) and lacked the recognizable cross-hatched pattern observed in the complex flagella of *S. meliloti *(Fig. 3f) [9,24,26,48] and *R. lupini *[40]. It is possible that the surface of the *R. leguminosarum *filaments lacks helical perturbations or the perturbations are not as prominent as those of the complex filaments of the other soil bacteria.

### Transcription of *R. leguminosarum fla *genes

Previous transcriptional studies in our lab using *gusA *fusions demonstrated that for both VF39SM and 3841, *flaA, flaC*, and *flaD *have the highest expression (2376 Miller Units (MU) to 6516 MU) while minimal expression (68 MU to 542 MU) was observed for *flaE, flaH*, and *flaG *[49]. The gene fusion for *flaB *reported in that paper was made in a different vector, pFAJ1701, so comparisons of *flaB *expression to that of the other flagellins were not valid. To place levels of *flaB *transcription in a proper context compared to the other *fla *genes, a new fusion to the *flaB *promoter was made in pFus1 (see methods) and gene expression of *flaB *was measured at 2529 ± 11 MU in 3841 and 4279 ± 466 in VF39SM. These results suggest that *flaA*, *flaB, flaC*, and *flaD *are the major flagellin subunits of *R. leguminosarum *while *flaE, flaH*, and *flaG *play minor roles. However, the presence of post-transcriptional regulation in flagellin biosynthesis cannot be precluded; hence, we performed mutational analysis. We have constructed strains with individual mutations in the seven flagellin genes and two multiple *fla *mutants (*flaB/C/D*^- ^and *flaA/B/C/D*^-^) for both strains VF39SM and 3841. The resulting mutants were examined for motility defects, using swimming and swarming assays, and morphological defects, using transmission electron microscopy.

### Motility assays and electron microscopy of wildtype and *fla *mutant strains

The swimming and swarming properties of the wildtype and *fla *mutant strains are summarized in Table 2. To account for the motility phenotypes of the mutant strains, we determined the effect of mutating the flagellin genes on the structure of the flagellar filament. In general, the flagellar filaments of all the individual flagellin mutants appeared to have normal fine structure and the width of the filament (except VF39SM *flaD*, which we describe below) was nearly identical to that of the wildtype.

**Table 2:** Properties of *R. leguminosarum *wildtype and flagellin mutants

Strain/Mutant	Effective Fla subunit(s)	Swimming diameter*	Swarming ability*	Filament Morphology†
Strain 3841	ABCDEHG	100	+++	Normal (4.7 ± 0.5um; n = 8)
3841 *flaA*^-^	BCDEHG	8	-	Almost all cells are non-flagellated; only one cell with very thin, short appendage
3841 *flaB*^-^	ACDEHG	47	+	Truncated (2.2 ± 0.5um; n = 6)
3841 *flaC*^-^	ABDEHG	30	++	ND
3841 *flaD*^-^	ABCEHG	87	+++	ND
3841 *flaE*^-^	ABCDHG	39	++++	Truncated (3.4 ± 0.3 um; n = 5)
3841 *flaH*^-^	ABCDEG	54	+++	Truncated (2.4 ± 0.6 um; n = 12)
3841 *flaG*^-^	ABCDEH	96	++	ND
3841 *flaB/C/D*^-^	AEHG	26	+	Truncated (1.9 ± 0.6 um; n = 13)
3841 *flaA/B/C/D*^-^	EHG	-	-	ND
				
Strain VF39SM	ABCDEHG	100	+++++	Normal (5.1 ± 0.5 um; n = 13)
VF39SM *flaA*^-^	BCDEHG	-	-	No flagella
VF39SM *flaB*^-^	ACDEHG	41	++	Truncated (1.6 ± 0.5 um; n = 6); reduced number of filaments (1-2 filaments/cell)
VF39SM *flaC*^-^	ABDEHG	49	++	Truncated (2.1 ± 0.5 um; n = 9); reduced number of filaments (1-2 filaments/cell)
VF39SM *flaD*^-^	ABCEHG	85	++++	Normal number and length; thinner filaments
VF39SM *flaE*^-^	ABCDHG	92	++++	Normal
VF39SM *flaH*^-^	ABCDEG	97	+++++	Normal
VF39SM *flaG*^-^	ABCDEH	100	+++	Normal; slightly reduced number of filaments
VF39SM *flaB/C/D*^-^	AEHG	25	+	Truncated (1.6 ± 0.3 um; n = 13); reduced number of filaments (1-2 filaments/cell)
VF39SM *flaA/B/C/D*^-^	EHG	-	-	No flagella

*Percentage relative to wildtype swimming diameter. Means of at least two replicates. (-) means non-motile; † As observed by TEM; ND means not determined; values in parenthesis refer to the average length of a flagellar filament ± standard deviation. The lengths of the flagella formed by the *fla *mutants are significantly different from the flagella formed by the wildtype strain (P < 0.0001).

**Figure 4:**
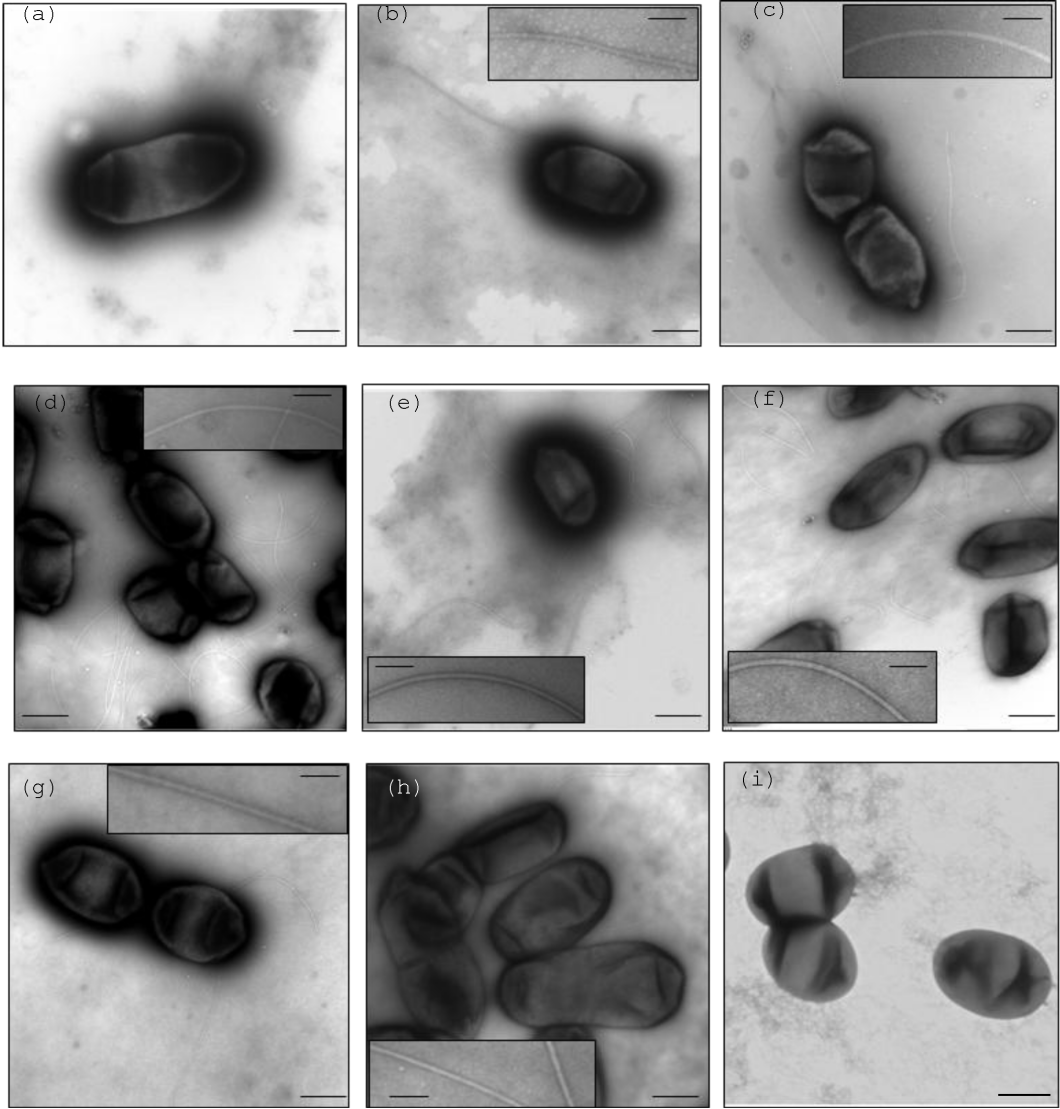
**Electron micrographs of *R. leguminosarum *VF39SM *fla *mutants stained with uranyl acetate**. Inset pictures show the flagellar filaments at higher magnification. (a) *flaA*^- ^(b) *flaB*^- ^(c) *flaC*^- ^(d) *flaD*^- ^(e) *flaE*^- ^(f) *flaH*^- ^(g) *flaG*^- ^(h) *flaB/C/D*^- ^(i) *flaA/B/C/D*^-^. Bars: 500 nm for cells with flagella; 100 nm for inset pictures.

**Figure 5:**
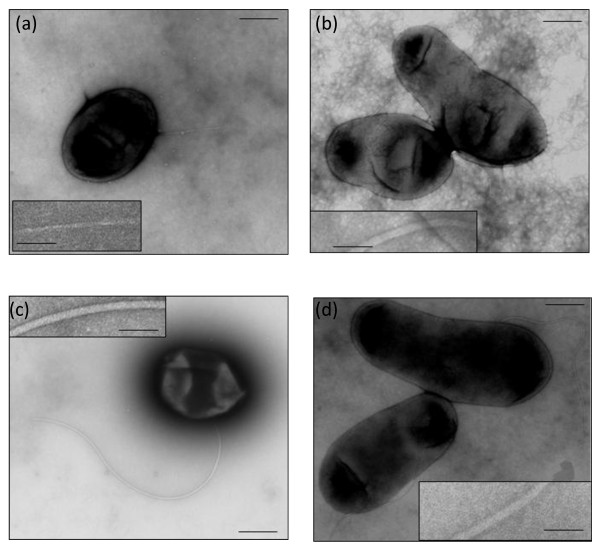
**Electron micrographs of *R. leguminosarum *3841 *fla *mutants stained with uranyl acetate**. Inset pictures show the flagellar filaments at higher magnification. (a) *flaA*^- ^(b) *flaB*^- ^(c) *flaE*^- ^(d) *flaH*^- ^Bars: 500 nm for cells with flagella; 100 nm for inset pictures.

The swimming motility of the 3841 *flaA *mutant was significantly reduced while the VF39SM *flaA *mutant was non-motile on swimming plates. Complementation of 3841 *flaA *and VF39SM *flaA *mutants with pBBRMCS1-MCS5::*flaA *completely restored swimming motility, confirming that swimming defects were due to loss of *flaA*. Both of the *flaA *mutants were also unable to swarm. The VF39SM *flaA *mutant strain was non-flagellated (Fig. 4a). Most of the 3841 *flaA *mutants observed by TEM were non-flagellated. Only one cell was observed to possess a very thin and short appendage (Fig. 5). Individual mutations in *flaB *for both 3841 and VF39SM, and *flaC *for VF39SM resulted in a reduced number of flagella and shorter filaments (Fig. 4b and 4c; Fig. 5), which could account for the considerable reduction in swimming and swarming motility (Table 2). The lengths of the flagellar filaments formed by the VF39SM *flaB and *VF39SM *flaC *mutants were reduced to around half of the wildtype flagellum. Mutation of *flaB *in 3841 also resulted in the synthesis of shorter filaments, exhibiting an average length of 2.2 μm. In terms of the number of filaments formed, almost all of the VF39SM *flaB*^- ^and VF39SM *flaC*^- ^cells observed exhibited only one flagellum per cell compared with the 4-7 flagella formed by the wildtype strain. Multiple mutations in *flaA/B/C/D *for both 3841 and VF39SM (Fig. 4i) resulted in non-flagellated and consequently non-motile strains. Complementation of the 3841 *flaA/B/C/D ^- ^*strain with cosmid 976 [50], which was shown by hybridization to carry *flaA, flaB, flaC*, and *flaD*, restored swimming and swarming motility to near wildtype levels (data not shown). The VF39SM *flaE *(Fig. 4e), *flaH*, and *flaG *mutants (Fig. 4f and 4g) exhibited normal flagellation while VF39SM *flaD *(Fig. 4d) displayed normal number and length of flagella, although the flagellar filaments were thinner along their entire length (average of 7 nm width). Also, individual mutations of *flaD, flaE, flaH*, and *flaG *did not significantly affect swimming and swarming motility in VF39SM (Table 3). A different phenotype was observed in 3841 *flaE *and *flaH *mutants, which exhibited truncated filaments (Fig. 5) and reduced swimming motility. The flagellar filaments formed by the 3841 *flaE*^- ^and 3841 *flaH^- ^*strains averaged 3.4 μm and 2.4 μm in length, respectively. Although the swimming motility of 3841 *flaE *and 3841 *flaH *mutant strains were reduced, the swarming motility was not significantly affected.

**Table 3:** Flagellin subunits and their relative abundance in *R. leguminosarum *wildtype strains based on tandem mass spectrometry analysis.

Flagellin subunit	Queries Matched	No. of unique peptides detected	Sequence coverage (%)	emPAI	Mascot score
A. 3841 wt lower band					

FlaB	21	4	42	5.85	856

FlaA	19	5	46	4.66	622

FlaC	12	2	41	1.46	401

B. 3841 wt upper band					

FlaB	22	4	37	4.05	741

FlaA	19	7	44	3.62	493

FlaC	13	3	31	1.23	288

A. VF39SM wt					

FlaB	36	5	43	8.28	1116

FlaA	24	8	46	6.68	748

FlaG	16	2	28	2.25	415

FlaC	18	2	29	1.72	469

FlaE	10	1	18	0.83	264

The motility assays and the filament morphologies demonstrate that FlaA is an essential flagellin subunit for *R. leguminosarum*. Mutation of *flaA *resulted in non-flagellated (for VF39SM) and consequently non-motile strains. It is possible that (at least for strain VF39SM), FlaA forms the proximal part of the filament, hence when FlaA is not synthesized, *R. leguminosarum *fails to assemble the distal part of the filaments using the other subunits synthesized. The major role of FlaA in filament assembly and function is similar to what has been reported in *S. meliloti*, *A. tumefaciens*, and *R. lupini *[5,6] . In all three species, mutation of *flaA *resulted in non-motile strains. However, unlike the non-flagellated VF39SM *flaA *mutant, strains of *S. meliloti, A. tumefaciens *and *R. lupini *with mutations in *flaA *were able to polymerize severely truncated filaments. Whereas FlaA is an essential subunit, it is not sufficient to assemble a fully functional flagellar filament as demonstrated in the *flaB/C/D *mutants. The *flaB/C/D *mutant strains exhibited shorter filaments and have reduced numbers of flagella (Table 2), which might have been assembled using FlaA and the other minor flagellin subunits (FlaE/H/G). In addition, the assembled filaments were not fully functional as demonstrated by the motility assays.

It is also apparent from our functional studies that both FlaB and FlaC are major components of the flagellar filament since mutation in each of the genes resulted in shorter filaments, reduced number of flagella, and consequently reduced motility. It is possible that FlaB and FlaC are located in the middle part of the filament, hence only the proximal part of the filament, composed of FlaA and possibly other minor subunits, is formed in the *flaB *and *flaC *mutants. Additionally, the reduction in the length and number of filaments in the *flaB *and *flaC *mutants may reflect an increase in the brittleness and fragility of the filament. Our claim that FlaA, FlaB, and FlaC are the major flagellins of VF39SM and 3841 is further supported by our gene expression studies which demonstrated high promoter activities for *flaA, flaB*, and *flaC*. It is also possible that FlaD contributes to the flagellar filament since the amount of *flaD *transcript was also high and the filaments formed by the VF39SM *flaD *mutant were thinner than the wildtype. The formation of thinner filaments also suggests that FlaD might be located along the entire length of the filament for VF39SM, thus the need for a high amount of *flaD *transcripts. However, it is remarkable that the swimming and swarming motility of the VF39SM *flaD *mutant are not impaired. A possible explanation could be that the width of the filament formed by the *flaD *mutant is still enough to support the normal function of the flagella. Contrary to the major roles of FlaA/B/C/D in VF39SM, FlaE, FlaH, and FlaG appear to be minor components of the flagellar filament as indicated by expression levels as measured in gene fusions, and by the subtle effects of their mutations on flagellar filament morphology and on motility. In 3841, FlaE and FlaH appeared to be important for swimming but not for swarming motility. Since the TEM images for the wildtype and *fla *mutant strains were obtained from vegetative cells, it would be interesting to observe the filaments formed by the swarm cells of 3841 *flaE *and 3841 *flaH *mutants.

### Tandem mass spectrometry analysis

**Figure 6:**
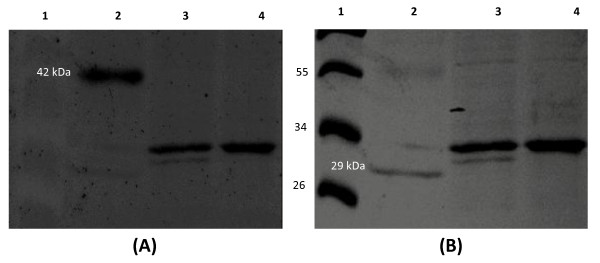
**Glycoprotein staining of *R. leguminosarum *flagellin proteins**. A. Pro-Q Emerald 300 stain. Lane 1-Molecular marker. Molecular masses (in kDa) are shown on the left of panel B; Lane 2-CandyCane glycoprotein molecular weight standard, 42kDa α1-Acid glycoprotein served as a positive control (shown in panel A) and a 29kDa-protein, carbonic anhydrase (shown in panel B) served as a negative control for glycosylation; Lane 3 - VF39SM; Lane 4 - 3841. B. Coomassie Brilliant Blue stain to demonstrate total proteins. Same sample arrangement as in panel A.

Flagellar samples were prepared from the wildtype strains and were run on SDS-PAGE. Immunoblots were prepared using a polyclonal flagellar antibody. However, due to the similar size of all seven flagellins (31-36kDa), we failed to resolve all subunits (Additional file 3). Thus, we decided to perform tandem mass spectrometry analysis to identify the flagellin subunits that are incorporated by the wildtype strains into flagellar filaments. We frequently observed two adjacent bands in the protein gel for both 3841 and VF39SM (see fig. 6 for VF39SM). To determine the subunits present in each of the two bands, the bands were analyzed separately for 3841. For VF39SM, the two bands were pooled together. Using the mass spectrometry data, we were also able to estimate the relative abundance of the flagellin subunits using the emPAI values [43] . It has been shown in a previous study that the emPAI value is directly proportional to protein content [44] and this parameter has been utilized in determining the relative abundance of a number of proteins [51-54]. The emPAI value provides an easy estimate of protein abundance since it is automatically generated using the Mascot program.

The locations of the flagellin peptides detected in the flagellar preparations are indicated in Fig. 1 and 2. Only FlaA, FlaB, and FlaC peptides were detected in the flagellar preparation for strain 3841 (for both the lower and the upper bands; Table 3) with sequence coverage ranging from 31% to 46%. These three subunits also comprised the majority of the flagellin subunits detected in VF39SM (Table 3). FlaE and FlaG comprised a small fraction of the flagellin subunits detected in the VF39SM wt strain. The sequence coverage for the flagellin subunits detected in VF39SM ranged from 18% to 46%. The results obtained from the MS/MS analysis indicate that at least three flagellin subunits (FlaA/B/C) are incorporated into the functional flagellar filament of strain 3841 while VF39SM polymerizes five flagellins (FlaA/B/C/E/G) into its flagellar filament. The consistently shorter flagellar filaments formed by the flagellin mutants (VF39SM/3841 *flaB *and *flaC *mutants) and the absence of flagellar filaments in VF39SM *flaA *mutants and nearly all cells of 3841 *flaA*^- ^also suggest that the major subunits (FlaA, FlaB, and FlaC), at least, are present in the complete flagella that are assembled.

Peptides for FlaD, FlaE, FlaH, and FlaG were not detected in the flagellar preparation for 3841 while FlaD peptides were not detected in VF39SM. The absence of the flagellin subunits could be due to the following reasons: (a) they are not synthesized under the conditions tested; (b) the subunits are synthesized but at a very low concentration, hence they remained undetected; and/or (c) the flagellin subunits are highly unstable. For strain 3841, mutation of *flaE *and *flaH *resulted in a reduction in swimming motility, suggesting that these subunits probably contribute to the flagellar filament. However, FlaE and FlaH peptides were not detected in the wildtype flagellar preparations, indicating that these peptides may not be stable under the conditions used.

### Glycosylation of flagellin subunits

We observed that for strain 3841, both the upper and the lower bands on the protein gel contained the same set of flagellin subunits (FlaA, FlaB, and FlaC) (Table 3). The molecular masses (around 35kDa; Additional file 3) of the bands observed on the gel also appeared to be higher than the predicted molecular masses (31kDa) for FlaA and FlaB. This suggests that at least FlaA and FlaB may have undergone post-translational modification, resulting in a higher molecular weight and subsequently slower migration in the protein gel.

Analysis of the flagellin amino acid sequences of *R. leguminosarum *(Fig. 1 &2) revealed the presence of two to four putative glycosylation signals (N-X-S/T, where X is any amino acid except proline) [55]. The MS/MS spectral data for the identified peptides containing the glyosylation signal were also analyzed for the presence of glycosylation, based on the presence of peaks (m/z) corresponding to different types of glycosylation (Additional file 4 shows a sample of a MS/MS spectrum). However, we have not identified any potential glycosylation for these peptides which may be attributed to the lability of this modification [56,57]. Also, sequence coverage only ranged from 18% to 46% (Fig. 1 and 2) and peptides at the C-termini of the flagellin subunits were not detected. The C-terminus contains a common glycosylation site for the *R. leguminosarum *flagellin subunits but these glycosylations were not detected in the MS/MS analysis, which could be due to the above reason. Thus, we performed glycoprotein staining to determine if the flagellins are post-translationally modified by glycosylation. We observed positive staining for the flagellins of both VF39SM and 3841 suggesting that these flagellins are glycosylated (Fig. 6). We were unable to determine which flagellins are glycosylated because the seven flagellins were not separated on the protein gel. Glycosylation of flagellins has been reported in a number of animal and plant pathogens including *Campylobacter jejuni *[56,57], *Helicobacter pylori *[57,58]*, Pseudomonas aeruginosa *[59,60], *Pseudomonas syringae *[61,62]*, Listeria monocytogenes *[63,64], *A. tumefaciens *[6]*, Acidovorax avenae *[65], as well as in the nitrogen-fixing bacterium *Azosprillum brasilense *[66]. It has been suggested that glycosylation may play a role in flagellar filament assembly and in pathogenesis [67,68]. In soil bacteria, it may also function in the attachment of bacteria to the plant roots [66], and in avoiding recognition by the host plant [61].

## Conclusions

In this study, we were able to clarify the roles of the seven flagellin subunits in the assembly of the flagellar filament in *R. leguminosarum*. Taken altogether, our results indicate that FlaA is an essential subunit, but that it is not enough to assemble a fully functional flagellar filament. FlaB and FlaC are major components of the filament while FlaD, FlaE, FlaH, and FlaG are only minor components. To assemble a fully functional filament, at least three (FlaA, FlaB, and FlaC) and five (FlaA, FlaB, FlaC, FlaE, and FlaG) flagellin subunits should be synthesized by 3841 and VF39SM, respectively. There were no substantial differences in the requirements for individual flagellins in swimming vs. swarming motility. The flagellins of 3841 and VF39SM are possibly modified by glycosylation.

## List of Abbreviations

MS: mass spectrometry; MS/MS: tandem mass spectrometry; LC: liquid chromatography; PBS: phosphate-buffered saline; emPAI: exponentially modified protein abundance index; TEM: transmission electron microscopy; SDS-PAGE: Sodium-dodecyl sulfate polyacrylamide gel electrophoresis.

## Authors' contributions

DDT was involved in the design of the study and in carrying out the experiments. DDT also prepared the draft for the manuscript. DEB and KLD were involved in conducting the experiments, which included construction of the mutants and *gusA *fusion strains and *gusA *assays. SFK was involved in the TEM work for the wildtype strains and some VF39SM mutants, and has been involved in revising the manuscript. MFK participated in interpreting the MS/MS results. MFH conceived the study, supervised the experiments, and was involved in writing and finalizing the manuscript. All authors read and approved the final manuscript.

## Supplementary Material

Additional file 1 **Sequences of primers used to PCR amplify flagellin genes**. Table showing PCR primer sequences for all PCR work discussed in the paper. Click here for file

Additional file 2 **Details of flagellin gene mutations in *R. leguminosarum *strains 3841 and VF39SM**. Table giving complete description of fragments and cassettes used in construction of all the mutants described in the paper. Click here for file

Additional file 3 **Immunoblot using an anti-flagellar antibody against flagellar preparations of *R. leguminosarum***. Figure showing western blot of flagellar preparations of wild type and mutant strains. Click here for file

Additional file 4 **MS/MS spectrum of one tryptic peptide from the data set for VF39SM**. Figure showing a Mass Spectrum of a peptide from the tryptic digest of VF39SM flagellar proteins. Click here for file
