# Supplementary material for: Characterization and functional analysis of seven flagellin genes in Rhizobium leguminosarum bv. viciae. Characterization of R. leguminosarum flagellins
Source: BMC Microbiol. 2010 Aug 17;10:219. doi: 10.1186/1471-2180-10-219 (PMC2936354; doi:10.1186/1471-2180-10-219)
Supplement: Additional file 1 — Sequences of primers used to PCR amplify flagellin genes. Table showing PCR primer sequences for all PCR work discussed in the paper. [file 1471-2180-10-219-S1.doc]

**Additional File 1**. Sequences of primers used to PCR amplify flagellin genes.

| Primer | Sequence (from 5’ to 3’) |
| --- | --- |
| *Primers (5’ to 3’)* |  |
| *flaA* forward primer | GCCTGTTTCTGGTATTCG |
| *flaA* reverse primer | GAAGGAAGCCGACTGAGC |
| *flaB* forward primer | AGGAAGAAATCGGTCAGC |
| *flaB* reverse primer | GAGAAGGAAGCCGATTGC |
|  |  |
| *flaC* forward primer | AAGAAATCAGCCAGTTGC |
| *flaC* reverse primer | GACCAGTAGGACGCAT |
| *flaD* forward primer | CTTATCGGCACATCATC |
| *flaD* reverse primer | CGCTTCCGTCATTACAG |
| *flaE* forward primer | TTGATAGTCAGTTTACC |
| *flaE* reverse primer | TTCGGCAATGAGATAGG |
| *flaH* forward primer | GCGAAGAACGGTATCCTG |
| *flaH* reverse primer | GGTATAAGGCGTGTCTGC |
| *flaG* forward primer | ACACGCTCAGGCAAAATC |
| *flaG* reverse primer | CATGGCTCAAACACTGCT |
