# Supplementary material for: Characterization and functional analysis of seven flagellin genes in Rhizobium leguminosarum bv. viciae. Characterization of R. leguminosarum flagellins
Source: BMC Microbiol. 2010 Aug 17;10:219. doi: 10.1186/1471-2180-10-219 (PMC2936354; doi:10.1186/1471-2180-10-219)
Supplement: Additional file 2 — Details of flagellin gene mutations in R. leguminosarum strains 3841 and VF39SM. Table giving complete description of fragments and cassettes used in construction of all the mutants described in the paper. [file 1471-2180-10-219-S2.doc]

**Additional file 2**. Details of flagellin gene mutations in *R. leguminosarum* strains 3841 and VF39SM.

| Gene mutated | Details of construction |
| --- | --- |
| **For 3841** |  |
| *flaA* | 2.8-kb *gusA-Nm^r^* cassette (CAS-GNm) from pCRS530 inserted into *flaA*  ORF using a *Sal*I site (cuts *flaA* at positions 220, 307, and 781) |
| *flaB* | Spectinomycin-resistance cassette from pHP45’Ω (*Xma*I) inserted into *flaB* ORF using *Age*I site (cuts *flaB* at position 583) |
| *flaC* | 2.8-kb *gusA-Nm^r^* cassette (CAS-GNm) from pCRS530 inserted into *flaC*  ORF using a *Sal*I site (cuts *flaC* at positions 220, 307, and 784) |
| *flaD* | 2.8-kb *gusA-Nm^r^* cassette (CAS-GNm) from pCRS530 inserted into *flaD*  ORF using a *Not*I site (cuts *flaD* at position 23) |
| *flaE* | 2.8-kb *gusA-Nm^r^* cassette (CAS-GNm) from pCRS530 inserted into *flaE*  ORF using a *Sal*I site (cuts *flaE* at positions 262, 307, and 645) |
| *flaH* | Km-resistance cassette from pBSL99 (*BamH*I) inserted into *flaH* ORF using a *Bcl*I site (cuts *flaH* at position 743) |
| *flaG* | Tetracycline-resistance cassette from pHP45-Tc (*Sma*I) inserted into *flaG* ORF using an *Age*I site (cuts *flaG* at positions 535 and 637) |
|  |  |
| **For VF39SM** |  |
| *flaA* | 2.8-kb *gusA-Nm^r^* cassette (CAS-GNm) from pCRS530 inserted into *flaA*  ORF using a *Sal*I site (same as 3841*flaA*; cuts *flaA* at positions 220, 307, and 781) |
| *flaB* | Spectinomycin-resistance cassette from pHP45’Ω (*Xma*I) inserted into *flaB* ORF using *Age*I site (same as *3841flaB;* cuts *flaB* at position 583) |
| *flaC* | 2.8-kb *gusA-Nm^r^* cassette (CAS-GNm) from pCRS530 inserted into *flaC*  ORF using a *Sal*I site (same as 3841 *flaC;* cuts *flaC* at positions 220, 307, and 784) |
| *flaD* | Construct with mutated *flaD* gene from 3841 (described above) used for gene replacement in VF39SM |
| *flaE* | 2.8-kb *gusA-Nm^r^* cassette (CAS-GNm) from pCRS530 inserted into *flaE*  ORF using a *Sal*I site (cuts *flaD* at positions 260, 598, and 643) |
| *flaH* | Construct containing mutated 3841 *flaH* gene (described above) used for gene replacement in VF39SM |
| *flaG* | Construct containing mutated 3841 *flaG* gene (described above) used for gene replacement in VF39SM |
