# Supplementary material for: Characterization and functional analysis of seven flagellin genes in Rhizobium leguminosarum bv. viciae. Characterization of R. leguminosarum flagellins
Source: BMC Microbiol. 2010 Aug 17;10:219. doi: 10.1186/1471-2180-10-219 (PMC2936354; doi:10.1186/1471-2180-10-219)
Supplement: Additional file 3 — Immunoblot using an anti-flagellar antibody against flagellar preparations of R. leguminosarum. Figure showing western blot of flagellar preparations of wild type and mutant strains. [file 1471-2180-10-219-S3.pdf]

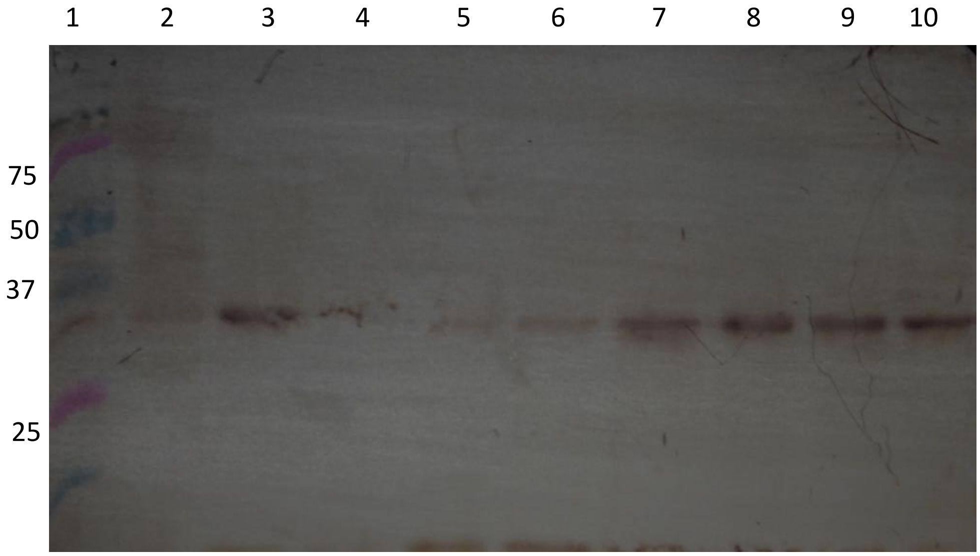


Fig.  S2. Immunoblot using an anti-flagellar antibody against flagellar preparations of *R. leguminosarum*.  Lane 1 – Molecular marker (molecular masses, in kDa, are on the left); Lane 2- empty; Lane 3- 3841 wildtype; Lane 4 – 3841*flaABCD*$^{-}$(no band was observed); Lane 5- 3841*flaA*$^{-}$; Lane 6- 3841*flaB*$^{-}$; Lane 7- 3841*flaC*$^{-}$; Lane 8- 3841*flaD*$^{-}$; Lane 9- 3841*flaE*$^{-}$; Lane 10- 3841*flaH*$^{-}$.
