# Supplementary material for: Characterization and functional analysis of seven flagellin genes in Rhizobium leguminosarum bv. viciae. Characterization of R. leguminosarum flagellins
Source: BMC Microbiol. 2010 Aug 17;10:219. doi: 10.1186/1471-2180-10-219 (PMC2936354; doi:10.1186/1471-2180-10-219)
Supplement: Additional file 4 — MS/MS spectrum of one tryptic peptide from the data set for VF39SM. Figure showing a Mass Spectrum of a peptide from the tryptic digest of VF39SM flagellar proteins. [file 1471-2180-10-219-S4.pdf]

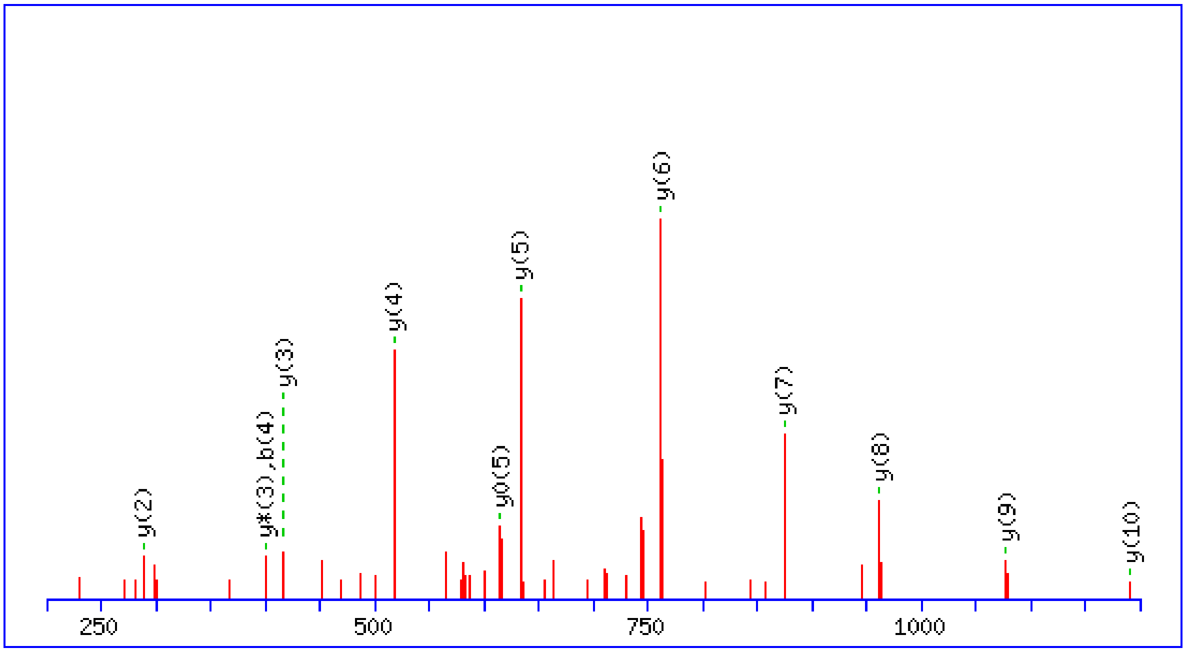

Fig. S1. Sample of a MS/MS spectrum from the data set for VF39SM. This is a MS/MS fragmentation of the peptide GINDSLEDTQNR found in FlaC (RL0720). Match to query 388 with a molecular mass of 1360.94. The observed molecular mass (1360.94) was close to the calculated monoisotopic mass (1360.62). No peaks corresponding to possible glycosylation of serine in the NDS glycosylation signal was observed.
